# Supplementary material for: Collagen Type XI Inhibits Lung Cancer-Associated Fibroblast Functions and Restrains the Integrin Binding Site Availability on Collagen Type I Matrix
Source: Int J Mol Sci. 2022 Oct 3;23(19):11722. doi: 10.3390/ijms231911722 (PMC9569509; doi:10.3390/ijms231911722)
Supplement: Supplementary file 1 [file ijms-23-11722-s001.zip › ijms-1893000-supplementary.pdf]

Supplementary Materials

Collagen type XI inhibits lung cancer-associated fibroblast functions and restraint the integrin binding site availability on collagen type I matrix

Cédric Zeltz, Maryam Khalil, Roya Navab and Ming-Sound Tsao

Supplementary Figures:

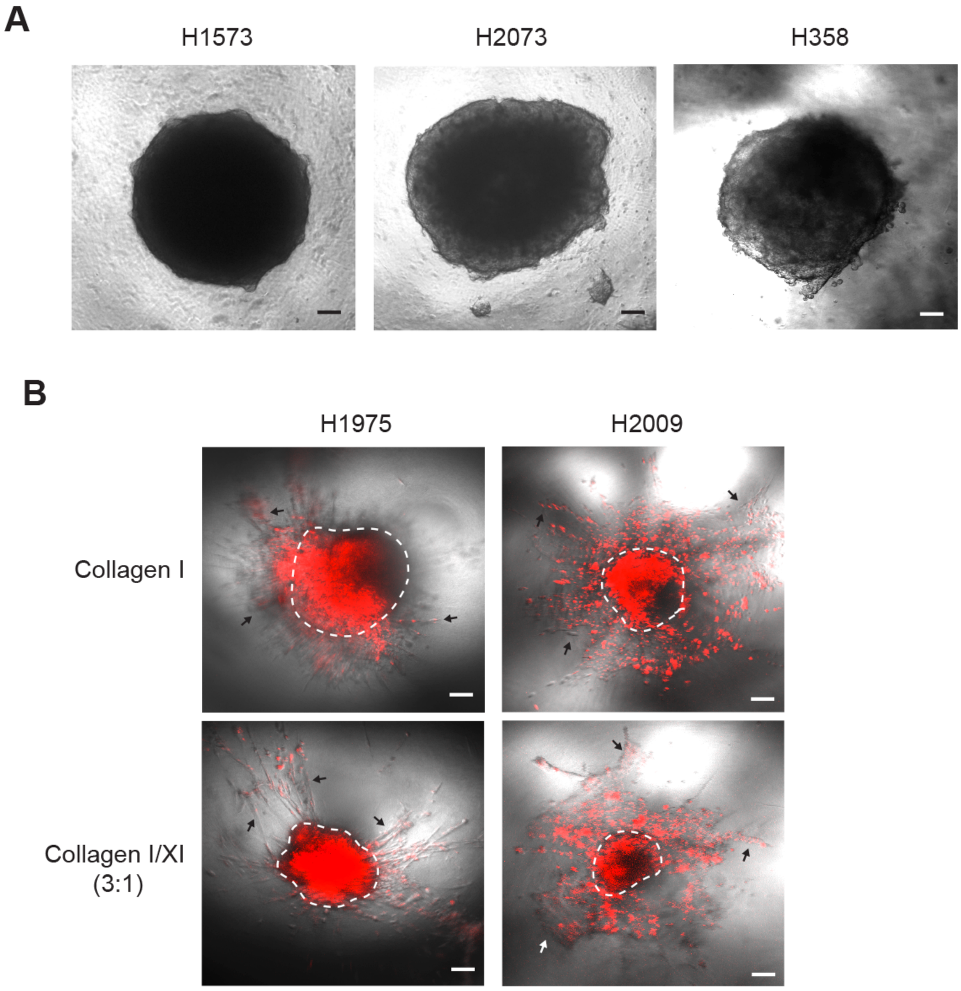

Supplementary Figure S1. A. H1573, H2073 and H358 lung tumor cells are slow invading in collagen type I matrix. Homospheroids of lung tumor cells were embedded in a matrix of collagen type I. The invasion into the collagen matrices was visualized for 5 days using confocal microscopy. Scale bars: 200 µm. **B.** Collagen XI does not inhibit CAF-independent H1975 and H2009 tumor cell invasion. Homospheroids of lung tumor cells (stained with a red dye) were embedded in a matrix of collagen type I or of a mix of collagen type I and collagen type XI at a 3:1 ratio. The invasion into the collagen matrices was visualized for 2 days using confocal microscopy. Scale bars: 300 µm. Arrows denote tracks in collagen matrix made by migrating CAFs. White dashes delimit the spheroids.

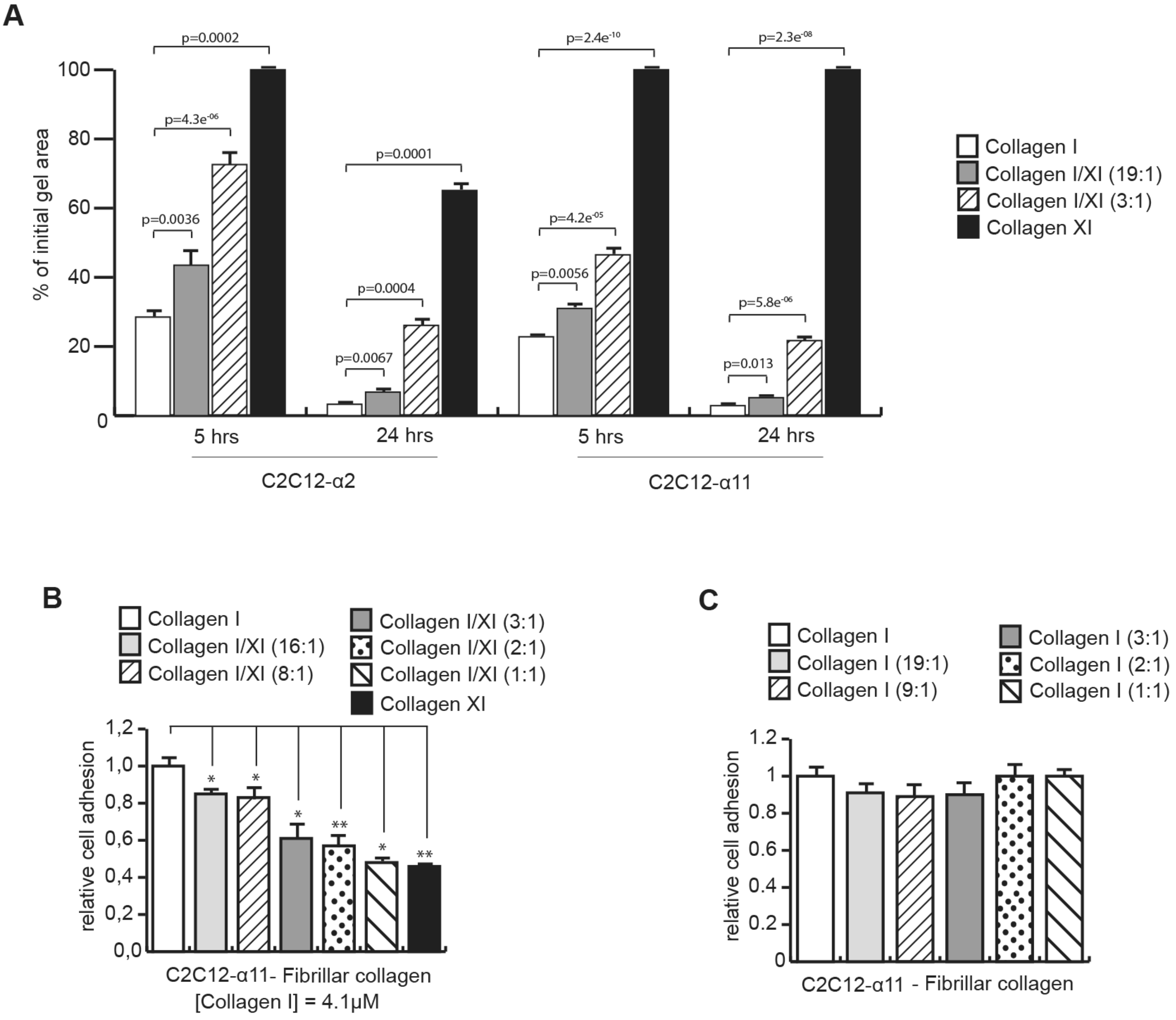

Supplementary Figure S2. A. Collagen type XI delays C2C12-mediated collagen matrix remodeling. C2C12 cells expressing either integrin α2 (C2C12-α2) or integrin α11 (C2C12-α11) were embedded in matrix of collagen type I, collagen type XI or of a mix of both collagens at a ratio of 19:1 and 3:1 (collagen type I:collagen type XI), and allowed to contract the matrix for 24 hours. Area of the collagen matrices has been measured at 5 and 24 hours. Statistics were performed using Student's test (mean±SD). **B.** Dose-effect of collagen type XI on inhibition of cell attachment to collagen type I. C2C12-α11 cells were seeded on fibrillar collagen type I, collagen type XI or a mix of both collagens at different ratios (collagen type I concentration was constant; 4.1µM) for 40 min. Cells were stained with Crystal violet and absorbance was red at 595nm. Statistics were performed using Mann-Whitney test (*, p=0.004998; **, p=0.004922, mean±SD). **C.** C2C12-α11 cell adhesion is not affected by the variation of collagen type I concentration. C2C12-α11 cells were seeded on fibrillar collagen type I at same concentrations than those used in the different ratios in the figure 4B. The concentrations of collagen type I used are indicated in the table 1.

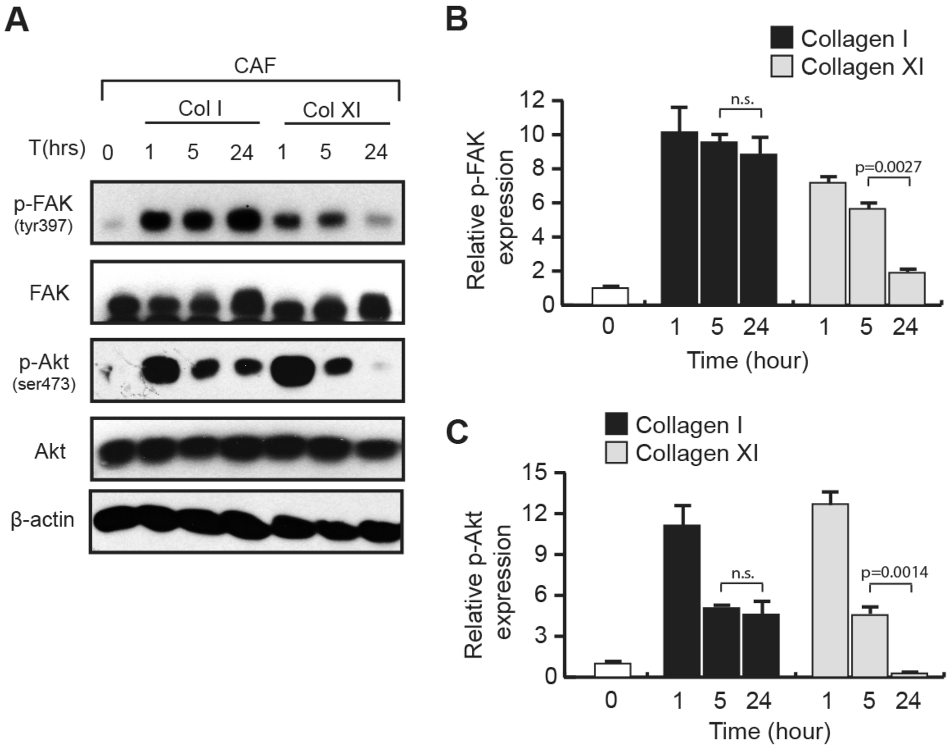

Supplementary Figure S3. Collagen type XI fails to sustain FAK and Akt phosphorylation in CAFs. **A.** Western blotting study of proteins extracted from CAFs seeded on collagen type I or collagen type XI at different time points. CAFs have been starved overnight before interacting with collagens. Phosphorylation of FAK and Akt has been analyzed. Phosphorylated FAK (**B**) and phosphorylated Akt (**C**) band intensity were quantified by densitometry analysis and normalized to their respective total protein. Statistics were performed using Student's test (mean±SD).
